# Trimethoprim resistance in Escherichia coli exhibits an allele-specific growth advantage

**DOI:** 10.1099/jmm.0.002021

**Published:** 2025-06-03

**Authors:** Alexandra Spencer, Qixiang Wong, Sophie T. Lawson, Holly Fry, Neha M. Ramchandani Ramchandani, Chris Harding, Judith Hall, Phillip D. Aldridge

**Affiliations:** 1 Biosciences Institute, Faculty of Medical Sciences, Newcastle University, Newcastle upon Tyne, UK; 2 School of Biomedical Sciences, Nutrition and Sport Sciences, Faculty of Medical Sciences, Newcastle University, Newcastle upon Tyne, UK; 3 Urology Department, Freeman Hospital, Newcastle upon Tyne Hospitals NHS Foundation Trust, Newcastle upon Tyne, UK; 4 Translational and Clinical Research Institute, Faculty of Medical Sciences, Newcastle University, Newcastle upon Tyne, UK

**Keywords:** antimicrobial resistance, *dfrA*, trimethoprim, urinary tract infections

## Abstract

**Introduction.** The antibiotic trimethoprim has been used to treat urinary tract infection (UTI) since ~1962. Alongside the nitrofurantoin, there are still justified reasons for trimethoprim use, especially in non-pregnant women. Trimethoprim resistance is commonly the result of acquiring the trimethoprim-insensitive dihydrofolate reductase gene: *dfrA*. Assessment of clinical *Escherichia coli* isolates from two clinical trials, AnTIC and ALTAR, identified carriage of two copies of *dfrA*.

**Hypothesis.** The hypothesis tested here was that dual *dfrA* carriage provided *E. coli* with a growth advantage.

**Methodology.** Two hundred and seventy-eight clinical isolates from AnTIC/ALTAR were assessed for *dfrA* carriage. Microplate-based growth assays assessed growth behaviour with and without 64 mg l^−1^ trimethoprim. Allelic replacement of *dfrA5* with five other alleles was also performed.

**Results.** One hundred and four isolates (37%) were identified to carry a total of 112 *dfrA* genes. Eight isolates (2.9%) carried two copies of *dfrA*. Comparison of *dfrA*^+^ to dual *dfrA* carriage could be differentiated by their growth behaviour when exposed to trimethoprim but had comparable MIC (>512 mg l^−1^). Analysis of all *dfrA*^+^ isolates determined that the growth behaviour exhibited an allelic bias. Allelic replacement of *dfrA5* with *dfrA1*, *dfrA7*, *dfrA8*, *dfrA14* and *dfrA17* demonstrated that the growth behaviour was *dfrA* specific.

**Conclusion.** This analysis determined that the dual carriage of two *dfrA* alleles generated a growth advantage to *E. coli*. However, the growth behaviour was dictated by allele carriage and not specifically dual carriage, as single carriage isolates also possessed the identified phenotype. This data suggests that there is a potential clinical impact dictated by *dfrA* allele carriage that could improve clinical decisions on management strategies of UTI.

## Introduction

The antibiotic trimethoprim is a 2,4 diaminopyrimdine derivative developed in the 1940/50s, first used in humans in ~1962 [1]. In bacteria, trimethoprim competitively inhibits the enzyme dihydrofolate reductase (DHFR) that catalyses the conversion of dihydrofolate to tetrahydrofolate [2]. Tetrahydrofolate plays a central role as a one-carbon donor in nt and aa synthesis.

Trimethoprim has been used to treat a range of bacterial infections since its early introduction. However, in combination with sulphonamides, it has been a front-line treatment of urinary tract infections (UTIs) since the early 1990s [3,4]. At that time, it replaced *β*-lactams, such as amoxicillin, due to the rapid rise of *β*-lactam resistance. In 2015, the UK NICE guidelines were updated to encourage the use of nitrofurantoin alongside trimethoprim [5] as there are justified reasons, including resistance data, that trimethoprim is still in use.

The predominant bacterial pathogen associated with UTIs is *Escherichia coli* [6]. The gene *folA* encodes the *E. coli* DHFR that is susceptible to trimethoprim inhibition [2]. Resistance to trimethoprim (Tri^R^) can occur via several pathways in *E. coli* including upregulation of *folA* expression and mutation of the binding pocket, leading to the loss of trimethoprim binding and thus inhibition [7]. However, the most common route is via the acquisition of a plasmid or integron-encoded trimethoprim-insensitive DHFR by horizontal gene transfer [8]. The gene responsible for Tri^R^ is now known as *dfrA*, previously DhfrI [8].

Acquired resistance to trimethoprim has been noted ever since it was introduced as an antibiotic. Current data suggests that Tri^R^ is associated with 30–40% of *E. coli* isolates associated with UTIs. However, this proportion is dictated by geographical factors and local antibiotic usage patterns [4]. The simplicity of the viewpoint that a single gene, *dfrA*, is responsible for such a high rate of resistance reflects the ‘tip of an iceberg’ that is Tri^R^. A recent assessment of GenBank entries curated the DfrA family to include 47 alleles of the gene: *dfrA1* to *dfrA48* (*dfrA2* is not used) [8]. *dfrA* encodes protein variants with a size range of 157 to 195 aa. To further complicate the phylogeny, a related gene encoding a DHFR (*dfrB*) generates a smaller protein ~78 aa long. Historically, *dfrB* was first described as *dfr2*, hence why *dfrA2* is not used to prevent confusion [8]. Phylogenetic analysis argues that the majority of *dfrA* alleles have evolved from *dfrA1* and *dfrA12*. DfrA variants share between 38 and 70% similarity to *E. coli* FolA with a median of 55.8%. While there are a high number of *dfrA* alleles frequently found within clinical and environmental isolates of species such as *E. coli*, the common alleles include *dfrA1*, *dfrA5*, *dfrA7*, *dfrA12* and *dfrA17* [9].

Treatment of an uncomplicated UTI is usually a 3–5-day course of antibiotics such as trimethoprim or nitrofurantoin. However, in up to 25% of cases, patients will present with a second or third UTI within 6 to 12 months. Guidelines for the management of patients suffering from recurrent UTI include low-dose daily prophylactic antibiotics such as nitrofurantoin, cephalexin or trimethoprim. Two recent clinical trials explored the efficacy of antibiotic prophylaxis to manage recurrent UTIs. AnTIC aimed to determine the clinical effectiveness of prophylactic antibiotics to reduce UTI incidence in intermittent self-catheterizing patients. The outcome of AnTIC showed that prophylaxis antibiotics could indeed reduce the incidence of UTI by 1 per annum [9]. The second trial, ALTAR, aimed to determine that the non-antibiotic treatment methenamine hippurate in female recurrent UTI patients was not inferior to daily antibiotics. The outcome of ALTAR suggested that while the methenamine hippurate group had a marginally higher rate of UTI episodes compared with a prophylactic antibiotic group (1.38 versus 0.89 UTI episodes), the absolute difference, 0.49 episodes per year, was of limited clinical consequence [10]. Both trials included the use of trimethoprim in the antibiotic arms. We aimed to explore *dfrA* carriage within *E. coli* isolates taken from AnTIC and ALTAR participants. Genomic surveillance suggested that occasionally *E. coli* isolates carried two copies of *dfrA*. This led to the research question: is the carriage of two *dfrA* alleles beneficial to *E. coli*?

## Methods

### Strains and general microbiology

All clinical isolates used during this study are described in Table S1 (available in the online Supplementary Material), while strains and plasmid needed for allelic replacements are described in Table S2. All pre-experiment cultures of bacteria were grown in either LB for genetic manipulation or Mueller Hinton Broth (MHB) for microplate assays overnight at 37 °C with constant shaking at 160 r.p.m. Trimethoprim was used at declared concentrations. Final concentrations for other antibiotics included kanamycin 50 mg l^−1^, chloramphenicol 12.5 mg l^−1^ and spectinomycin 50 mg l^−1^.

### Growth assays

For standard batch culture growth assays, overnight cultures were inoculated into fresh MHB media with a starting OD600=0.02. Cultures were then incubated at 37 °C with constant shaking at 160 r.p.m. OD600 and viable counts were measured every 30 min for 420 min. For microplate-based growth assays, overnight cultures of respective strains were diluted 1:10,000 in MHB, without normalization as each strain was being compared with itself. Trimethoprim concentration gradients were prepared as 1:2 dilutions of a 2× stock in MHB medium using a 100 µl volume in each well. Prior to incubation, 100 µl of the 1:10,000 culture dilution was added to the trimethoprim+MHB solution. For MIC assays, an extended concentration range of trimethoprim was used (0.25 to 1,024 mg l^−1^). The suggested range for antibiotic susceptibility for trimethoprim is 0.03 to 128 mg l^−1^ [11]; however, this extended range is consistent with Sánchez-Osuna *et al.* [12]. The same principle was used for the screen where each strain was inoculated into four wells, two with no trimethoprim and two with a final concentration of 64 mg l^−1^. Microplates were then incubated in a BMG Fluostar microplate reader for ~22 h using kinetic measurement of absorbance at 600 nm every 410 s for 200 cycles. In between measurements, plates were shaken for 300 s in a double orbital manner. Desiccation was prevented by sealing the plate with a BreatheEAsy membrane.

### DNA sequencing

For Illumina sequencing, genomic DNA was isolated using the GENelute genomic DNA isolation kit (Sigma). For nanopore sequencing, a standard phenol:chloroform-based DNA extraction protocol was used. Lysis was achieved in a Tris-EDTA (TE: 10 mM Tris-Cl pH 8.0, 1 mM EDTA) buffer containing 0.6% SDS, 0.12 mg ml^−1^ proteinase K and 0.1 mg ml^−1^ RNase with incubation at 37 °C for 1 h. Two washes in an equal volume of ultrapure phenol:chloroform (Invitrogen) were used to extract DNA before precipitation with 2.5× volume isopropanol and a 70% ethanol wash step. All DNA was quantified using a QUBit system after DNA had been resuspended in TE buffer. Sequencing was performed at in-house facilities at either Newcastle University or Northumbria University.

### Bioinformatic analysis

Sequenced genomes were quality assessed and if necessary trimmed with TRIMMOMATIC [13]. All assemblies were performed *de novo* using UNICYCLER 0.4.8 either with just Illumina data or where available both Illumina and nanopore data [14]. Annotation of assembled genomes was performed using PROKKA 1.14.6 [15]. For analysis of *dfrA* carriage, an in-house blast+ 2.150.0 protein database was generated using all [strain].faa files from the annotation step. To identify DfrA carriage, four alleles were used, DfrA12 (CAA79767), DfrA36 (QBZ35659), DfrA1 (CAA25445) and DfrA7 (CAA41326), which represent the predicated ancestral sources of DfrA (DrfA1 and A12) and common/recently described variants (DfrA7 and A36). Outputs were curated from blastp into tabulated form for analysis. Phylogenetic analysis was performed from muscle 5.1 [16] alignments with maximum likelihood phylogenetic trees generated in mega 11 (https://www.megasoftware.net/) [17]. Multilocus sequence typing (MLST) was performed by extracting the MLST sequences from each genome and then performing a batch analysis of the data at PUBMLST.org. All analysis was performed using bash scripting through Terminal MacOS. All sequence alignments shown in the supplementary data were generated from muscle alignments using pyBoxshade (https://github.com/mdbaron42/pyBoxshade). Where necessary, data was analysed and graphically visualized using R. Fig. 3 specifically exploited the R packages ape and ggtree where the tree is a Newark output from mega 11 of a maximum likelihood tree. All sequence data has been deposited to the EBI-Nucleotide archive project numbers: for AnTIC-derived isolates, PRJEB39670; ALTAR short-read data, PRJEB85317; and ALTAR long-read data, PRJEB85595. Direct accession numbers to each dataset are provided in Table S1.

### Growth analysis and statistical tools

Growth analysis was performed in R using custom-built scripts available on request. The maximum growth rate was determined as previously described [5]. Lag analysis and area under the curve were calculated using custom-written scripts available on request. Where necessary, statistical tools embedded in R were used to determine the significance of datasets.

### Allelic replacement of *dfrA* alleles

Allelic replacement methodology has been described in detail previously [18]. All primers used are described in Table S3. Briefly, *dfrA5* was deleted with a chloramphenicol cassette derived from pWRG100 [19] with the assistance of pKD46-Kan [5] after cells had been grown in 0.1% arabinose from an OD600 of 0.1 to 0.6–0.8. Replacement of the Cm^R^ was achieved using CRISPR technology combining pCas9 [20] with pTRG-Cm [18]. DNA fragments flanking each *dfrA* allele from a donor strain were generated, and 1:50 dilutions of the pTRG-Cm/PCR transformations were plated out on kanamycin+spectinomycin plates and M9+trimethoprim (50 mg l^−1^) plates. Tri^R^ colonies from the M9 plates were used for downstream confirmation of successful recombination.

### Gene expression analysis

Quantitative PCR (qPCR) primers were designed for each target allele using Primer-blast hosted at https://ncbi.nlm.nih.gov and are provided in Table S3. RNA was extracted from strains grown in MHB without trimethoprim at 37 °C until an OD600 ~0.6 using the SV Total RNA isolation kit (Promega) with one further DNAase treatment after elution using TUBRO DNA Free (Invitrogen). cDNA was generated using the one-step LunaScript RT supermix kit (NEB). Reverse Transciptase qPCR (RT-qPCR) was performed in a ROTORGENE using QuantiNova SYBR Green PCR kit (QIAGEN) using a standard cycle programme. Transcript copy was determined using standard curves of genomic DNA from the same strain. Standard curves of genomic DNA assumed one copy of a *dfrA* gene were calculated based on genome size (including plasmid data) in tenfold steps from 300,000 to 30 copies. All data was analysed in Excel from Ct values extracted from the ROTOGENE software using a threshold of 0.25.

## Results

The isolates of *E. coli* used throughout this study originate from the clinical trials AnTIC and ALTAR [9,10]. The study protocol for both trials allowed for the isolation of *E. coli* from urine, if the diagnostic criteria were met, and from a perineal swab. A total of 278 (ALTAR: 191; AnTIC: 87) isolates were sequenced using a combination of short- and long-read technology (Table S1). Forty-seven of the urine-associated *E. coli* isolates sequenced from AnTIC have been previously described [21]. The other 40 included 8 perineal swabs and 32 urine-associated *E. coli* chosen randomly from the AnTIC strain collection. The criteria used to choose the 191 *E. coli* isolates from ALTAR included the following: (i) date matched swab and urine-associated isolates, (ii) 2+ temporal urine-associated isolates per patient and/or (iii) temporal perineal swab isolates that included a baseline *E. coli* isolate. This generated a dataset that represented 158 colon-derived and 120 urine-associated *E. coli* isolates, with a >98% providing a temporal snapshot of specific patients from both trials.

### Genome surveillance of *dfrA* carriage and diversity

After genome assembly and annotation, blastp was used to identify FolA and DfrA hits from an in-house blast database containing all annotated protein sequences for each genome. Eleven of 278 isolates had single aa changes in the predicted FolA sequence L28R (*n*=3), W30R (*n*=5) and P105R (*n*=3). The L28R substitutions were all from one patient in AnTIC on trimethoprim prophylaxis. Leucine 28 is known to play a key role in the binding of trimethoprim to FolA [7]. However, the impact of this specific substitution was not investigated further as the focus was *dfrA* carriage.

**Fig. 1.**
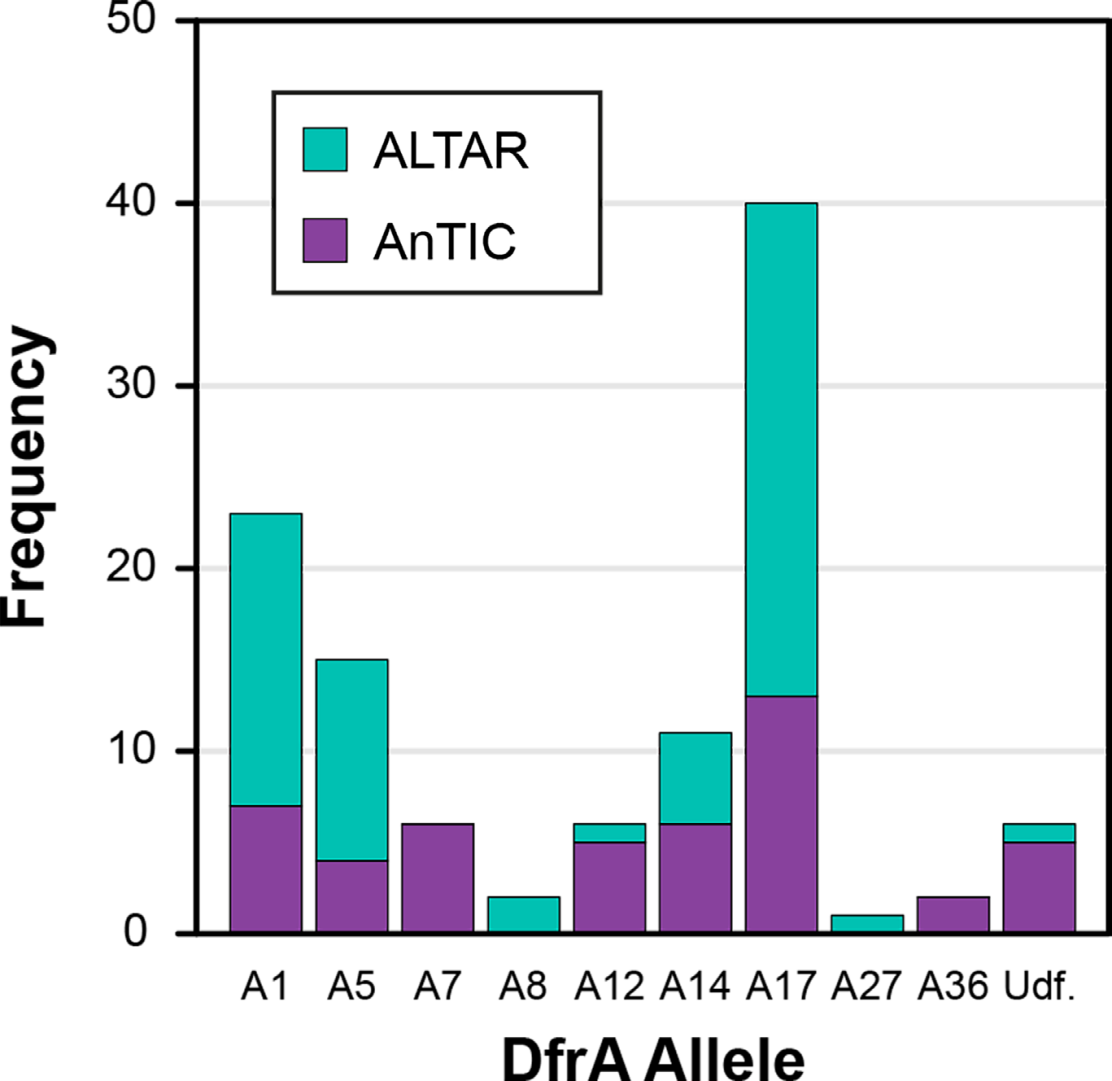
*dfrA* alleles identified from AnTIC and ALTAR *E. coli* isolates. Analysis shows the total number of each allele identified with each bar split between the trial source (see key for colour). Bioinformatics identified six alleles (Udf=undefined) that could not be grouped with known alleles by association alone. For this study, these were defined as Axx (*n*=5) and Ayy (*n*=1).

From 104 isolates, 112 DfrA protein hits were identified. Phylogenetic analysis identified nine known alleles to be represented in the AnTIC/ALTAR dataset and six hits that could not be defined by association (Fig. 1). At a low frequency, DfrA7 and DfrA36 were identified in AnTIC isolates, while DfrA8 and DfrA27 were unique in ALTAR isolates. Previous analysis suggests DfrA1, DfrA5, DfrA7, DfrA12 and DfrA17 are the common alleles [22,23]. The AnTIC/ALTAR dataset is consistent with this observation, with DfrA17 being the most frequently identified variant (Fig. 1). Interestingly, DfrA14, found 11 times, could also be defined in this category.

**Fig. 2.**
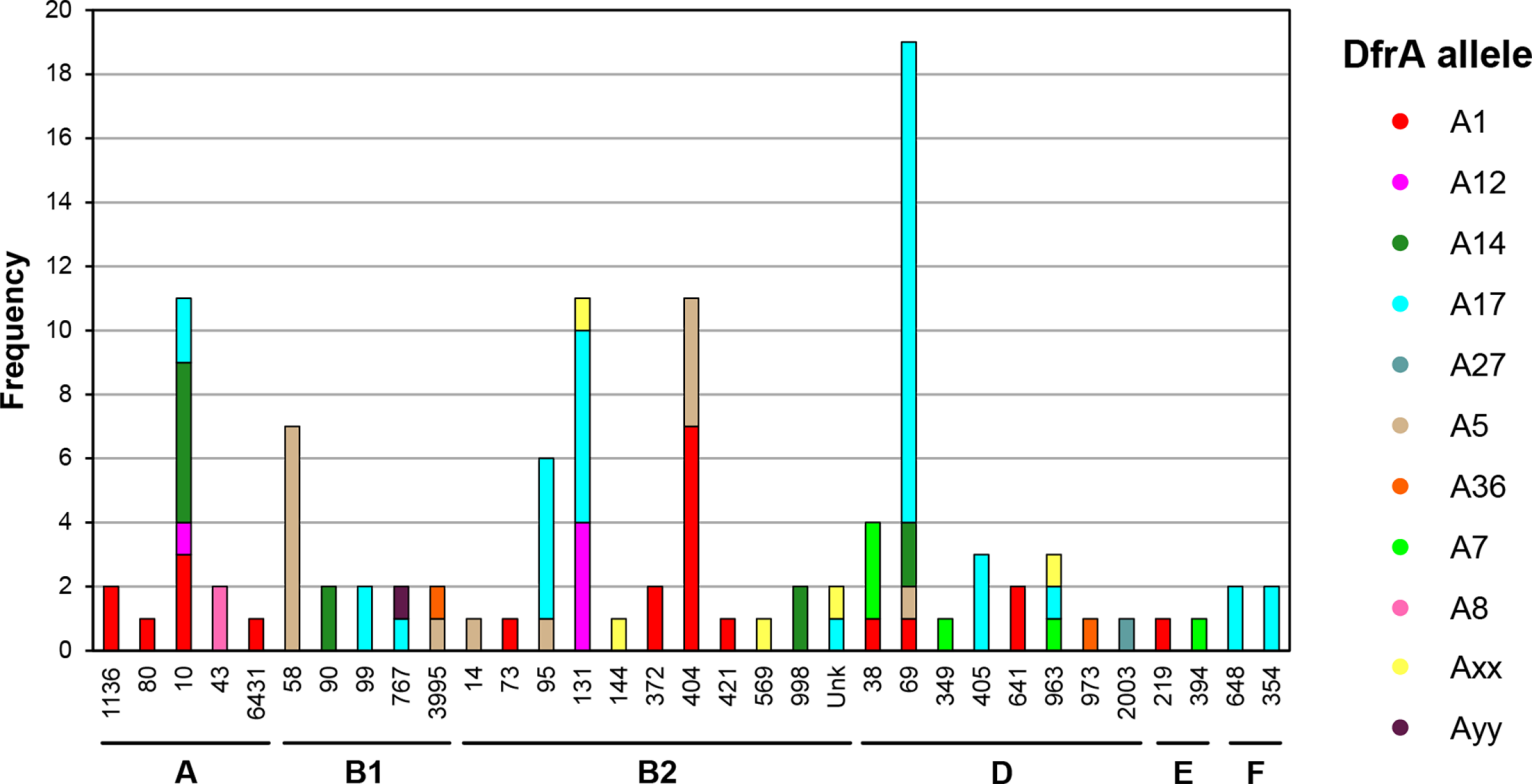
Diversity of *E. coli* isolates compared with known *dfrA* alleles. Bars represent the total number of each ST shown along the x-axis, while colours based on the figure key declare the *dfrA* allele. One strain could not be genotyped using the Achtman scheme. Phylogenetic analysis using concatenated MLST sequences defined the isolate of unknown genotype (Unk.) to be clade B2.

**Fig. 3.**
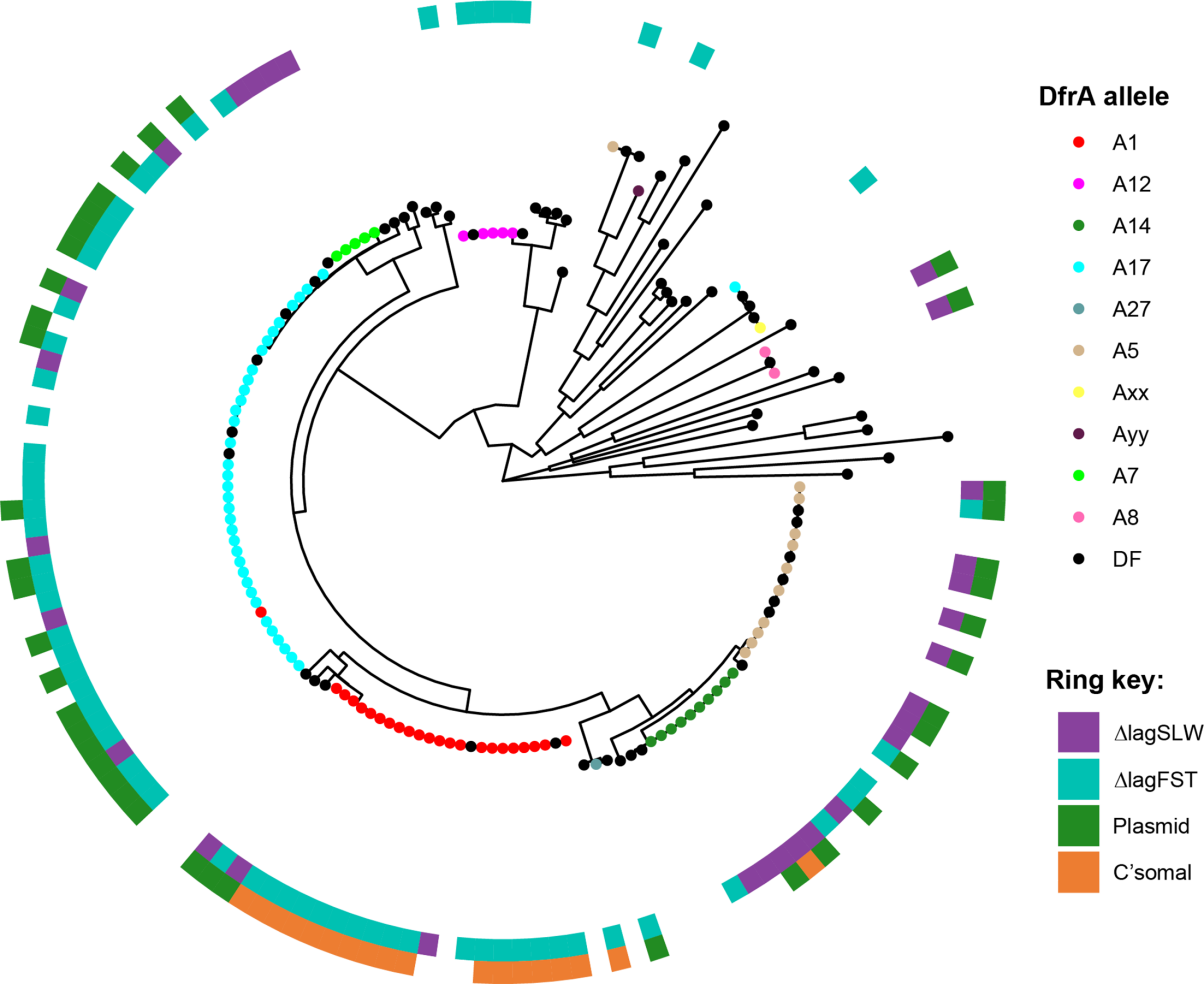
Phylogenetic analysis of *dfrA* alleles exemplifies the bias of ∆lag_SLW_ to *dfrA5* and *dfrA14* and how location, either on a plasmid or the chromosome, does not have a significant impact on the growth behaviour. Keys indicate colour use on phylogenetic tree and ring heatmaps. DF=Reference *dfrA* alleles taken from Ambrose and Hall [8]. Where a colour node on the tree is out of place indicates isolates that carry two *dfrA* alleles. The gaps in the outer ring are isolates where the quality of the genome assembly prevented definitive recognition of the *dfrA* allele being chromosomal or encoded on a plasmid.

Analysis of the genomes harbouring *dfrA* gene alleles, using the Achtman MLST scheme [24], defined 31 sequence types (STs) distributed across the clades of *E. coli* (Fig. 2). The majority of isolates are associated with clade B2 and D. STs 10, 58, 95, 131, 404 and 69 all had >5 isolates within the dataset. Stratification of *dfrA* alleles with respect to ST suggested a bias for specific STs and alleles. For example, ST58 was exclusively found to harbour *dfrA5*, and ST69 was predominantly associated with *dfrA17*. The most diversity, *n*=4 alleles, was observed for ST10 and ST69. *dfrA1* exhibited the broadest distribution amongst STs (Fig. 2). *dfrA1* is encoded within an integron predominantly found on the *E. coli* chromosome adjacent to the *glmS* locus, while the majority of other *dfrA* alleles were plasmid encoded (Fig. 3). This analysis demonstrates that there is a significant level of diversity with respect to *dfrA* carriage and strain phylogeny within the AnTIC/ALTAR dataset.

It was further noted that eight isolates (2.9%) carried two *dfrA* alleles: four isolates carried *dfrA1*/*dfrA5*, a *dfrA1*/*dfrA17* isolate, a *dfrA5/dfrA36* isolate and two isolates encoding *dfrA17* with an undefined *dfrA* (*dfrAxx*) allele. This observation is consistent with other surveillance studies [25], leading to the research question: does two copies provide any advantage?

### Growth behaviour of single and dual *dfrA* carriage

**Fig. 4.**
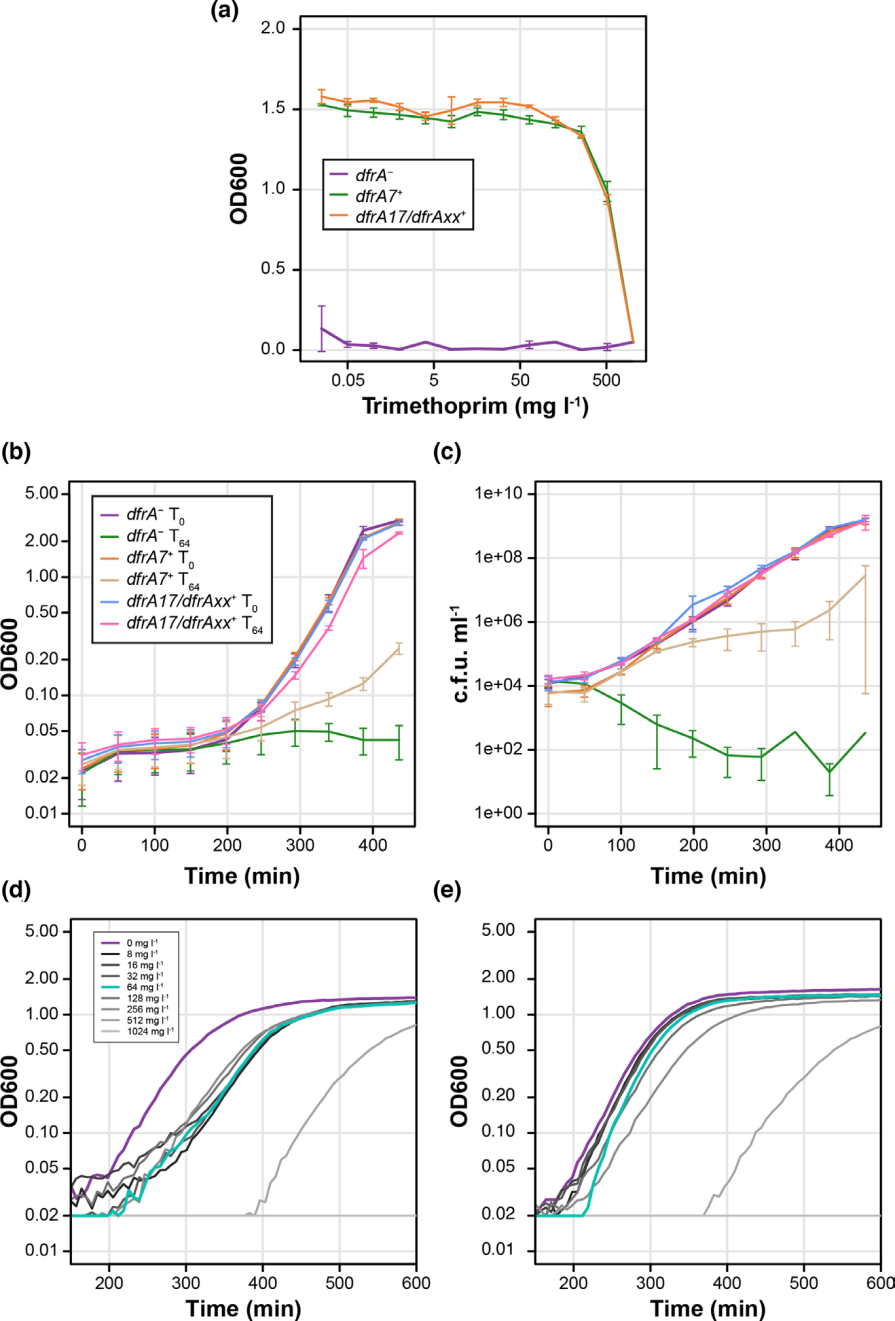
MIC and growth analysis of *dfrA*^-^, *dfrA7*^+^ and *dfrA17*/*dfrAxx*+ isolates. (**a**) MIC analysis suggests that there is no difference between the MIC for each Tri^R^ isolate. (**b**) Growth behaviour of each isolate when challenged with trimethoprim (T_64_) compared to no trimethoprim (T_0_) differentiates *dfrA7*^+^ from *dfrA17*/*dfrAxx*^+^. (**c**) Average viability data of strains monitored by OD in (**b**) presents a consistent picture. Microplate-based growth assays using a range of trimethoprim concentrations show that the growth behaviour of (**d**) *dfrA7*^+^ and (**e**) *dfrA17*/*dfrAxx*^+^ is independent of trimethoprim concentration. See keys for colour explanations; error bars are not shown in (d) and (e) for clarity. All data represents the average of a minimum of three independent biological repeats.

Three AnTIC isolates carrying *dfrA7* (*dfrA7*^+^), *dfrA17* and an undefined *dfrA* allele (*dfrA17*/*dfrAxx*^+^) and a Tri^S^ isolate (*dfrA*^-^) were chosen as case examples to address the advantage of double carriage. The choice of *dfrA17*/*dfrAxx*^+^ was based on the carriage of an undefined allele, making it an interesting case to study. Both Tri^R^ strains exhibited an MIC >512 mg l^−1^ (Fig. 4a). To determine whether the growth behaviour of these case examples reflected the MIC data, an intermediate concentration of 64 mg l^−1^, eight times lower than the MIC, was chosen. Monitoring growth and viability with and without 64 mg l^−1^ trimethoprim allowed *dfrA7*^+^ to be differentiated from *dfrA17/dfrAxx^+^* (Fig. 4b, c). The *dfrA7*^+^ strain exposed to 64 mg l^−1^ trimethoprim had a reduced growth rate and lower viability compared with the *dfrA17/dfrAxx^+^*strain. Furthermore, using a microplate-based growth assay, exposing *dfrA7*^+^ and *dfrA17/dfrAxx^+^* to trimethoprim concentrations between 8 and 1,024 mg l^−1^, defined the growth behaviour to be independent of concentration (Fig. 4d, e). This suggests that, based on these case examples, dual carriage *dfrA* alleles versus single carriage can be differentiated by their growth characteristics when exposed to trimethoprim.

### Screening all *dfrA*^+^ for growth behaviour in the presence of trimethoprim

The case examples suggested an advantage of carrying two *dfrA* alleles when exposed to trimethoprim, leading to all 104 isolates being screened for their growth behaviour. This would test the hypothesis that only carriers of two *dfrA* alleles will possess the faster growth phenotype. To differentiate between growth behaviours, the time taken to reach an OD600 threshold of 0.15 was calculated for no trimethoprim (T_NT_) and with trimethoprim (T_64_). A strain was declared as having the fast phenotype (∆lag_FST_), exhibited by *dfrA17/dfrAxx^+^*, if T_64_-T_NT_ was less than the doubling time plus 5 min (DT_nt+5_) of that strain grown with no trimethoprim. The addition of 5 min was used to account for the average sd of all doubling times measured without trimethoprim (Table S4). For strains defined as having a slow growth phenotype (∆lag_SLW_), similar to *dfrA7*^+^, T_64_-T_NT_ needed to be greater than DT_nt+5_ (Table S4).

The bioinformatic analysis identified five isolates carrying a protein with close similarity to DfrA4, which we defined as DfrAxx (Figs1^ 3^ and S1). However, based on the criteria of Ambrose and Hall [8], these alleles could not be defined as DfrA4. Analysis of the screening data showed that every isolate carrying just this gene was Tri^S^ (Table S4), with the only isolates showing Tri^R^ being the AnTIC case example *dfrA17/dfrAxx^+^* and UAN8530, an unrelated strain also carrying *dfrA17/dfrAxx^+^* (Table S4). This suggests that phenotypically the identified gene does not confer Tri^R^ and thus is not *dfrA*. However, the sixth undefined allele (here defined as *dfrAyy*) shares 65% identity to DfrA36, which would justify a new allele number being Tri^R^ possessing the ∆lag_FST_ phenotype (Table S4 and Figs1, 3  5a).

**Fig. 5.**
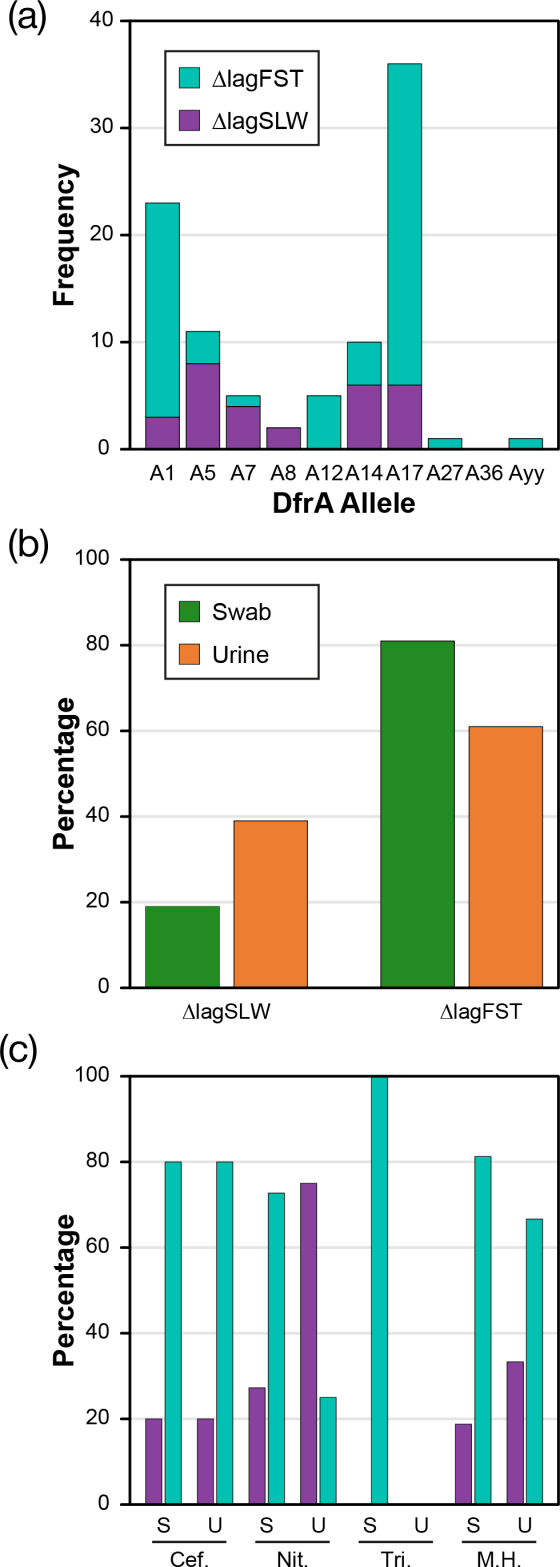
Distribution of ∆lag_SLW_ and ∆lag_FST_ phenotypes when correlated to (a) dfrA alleles derived from data incorporating both AnTIC and ALTAR isolates, (b) source of isolate from the ALTAR trial and (c) treatment given during the ALTAR trial.

In contrast to *dfrA17/dfrAxx^+^*, all other isolates carrying two *dfrA* alleles did indeed possess the ∆lag_FST_ phenotype. However, a significant number of single carriage isolates also did (Fig. 5a and Table S4). Furthermore, the distribution of ∆lag_FST_ and ∆lag_SLW_ in relation to the *dfrA* allele suggested that the observed phenotypes exhibited a given level of allele bias (Figs 1 and 5a). *dfrA1*, *A12* and *A17* were all associated predominantly with ∆lag_FST_ (Fig. 3). *dfrA5*, *dfrA7*, *dfrA8* and *dfrA14* exhibit a bias towards ∆lag_SLW_ (Fig. 3). This suggests that the identified difference in growth behaviour is not based on how many alleles an isolate encodes but which *dfrA* allele is encoded by any given isolate, exemplified by the case example *dfrA17*/*dfrAxx*^+^ itself.

### The clinical impact of the *dfrA* growth phenotype

The ALTAR isolates (*n*=191) provide an opportunity to stratify the growth behaviour data based on the source of isolate (urine or perineal swab) and primary treatment used during ALTAR. In relation to the source of isolate, the slow phenotype was more frequently associated with uro-associated isolates (Fisher exact test *P*=0.0029) (Fig. 5b). Furthermore, based on treatment (Fig. 5c), both phenotypes could be identified in isolates from patients on cefalexin, nitrofurantoin and methenamine hippurate. In contrast, patients taking trimethoprim prophylactically only had fast-growing Tri^R^ isolates associated with the perineal swabs. Analysis of patient outcome (incidence of UTI) showed that the slow phenotype of *dfrA5*, *dfrA8* and *dfrA14* was predominantly associated with patients recording no UTI during ALTAR. However, none were on trimethoprim, preventing a correlation to the impact of the phenotype on trimethoprim use.

### Is it *dfrA* or the strain that dictates the growth phenotype?

Analysis of the growth behaviour of Tri^R^ isolates suggests that, when exposed to trimethoprim, the observed phenotype is dependent on which *dfrA* allele a specific isolate encodes. Therefore, to determine if the *dfrA* allele itself is driving the observed growth behaviour, allelic replacement of the coding sequence of *dfrA5* in an ST58 clinical isolate was replaced with *dfrA1*, *dfrA7*, *dfrA8*, *dfrA14* and *dfrA17*.

nt sequence diversity between *dfrA5*, *dfrA1*, *dfrA8* and *dfrA14* meant that recombination occurred at the desired start codon position of *dfrA5*. During allelic replacements, a conserved 142 bp region from the *xerC* start codon to the annotated start codon of *dfrA5*, *dfrA7*, *dfrA12*, *dfrA14* and *dfrA17* was identified (Fig. S2). The coding capacity of this region exhibits strong similarity to N-terminal extensions often seen for annotated *dfrA7* (25 or 76 aa long) and *dfrA17* (25, 42 and 76 aa long) alleles (Fig. S3A). This homologous region restricted the options to generate the desired *dfrA5:dfrA7* and *dfrA5:dfrA17* recombinant isoforms. A recombinant *dfrA5:A17* generated an ORF encoding a 199 aa protein similar to 11 out of 41 isolates in the AnTIC/ALTAR dataset. This was defined as *dfrA5:A17_L_* (Fig. S3B). A second *dfrA5:A17* recombinant was successfully designed, generating a shorter 157 aa *dfrA17* protein *dfrA5:A17_S_*. For the *dfrA5:A7* recombinant, while the intended gene would encode a protein 182 aa long, the predicted ORF encodes an extended isoform 230 aa long (Fig. S3B).

All recombinants were assayed for their growth behaviour in comparison with the parental ST58 *dfrA5* isolate and the *dfrA7*^+^ and *dfrA17/dfrAxx*^+^ case examples (Fig. S4). To complement ∆lag analysis, the difference in the area under the curve (∆AUC) was also determined (Table 1). For *dfrA8* and *dfrA17*, the isoforms reflected their parental phenotypes, exhibiting a change in growth behaviour compared with the *dfrA5* parent. *dfrA5:A14* had very little change, which is consistent with these two alleles being closely related (Figs 1 and S4 and Table 1). Irrespective of the isoform of *dfrA17*, a fast phenotype was observed with a significant drop in ∆lag and ∆AUC compared with *dfrA5* (Table 1). This data suggests that the growth behaviour in response to trimethoprim is *dfrA* dependent.

**Table 1.** Growth characteristics of *dfrA5*:A(x) recombinants

*dfrA* gene	Parent phenotype	DT_MAX_*	∆Lag	∆AUC†	Isoform phenotype
*dfrA5*	Slow	17.8	37.5	61.7	–
*dfrA5:A1*	Fast	18.6	1041.1	172.7	Slow
*dfrA5:A7*	Slow	14.5	6.8	25.8	Fast
*dfrA5:A8*	Slow	13.6	117.8	167.2	Slow
*dfrA5:A14*	Slow	15.3	32.46	59.0	Slow
*dfrA5:A17_S_*	Fast	13.7	6.8	26	Fast
*dfrA5:A17_L_*	Fast	14.1	11.9	26.3	Fast
*dfrA7§*	Slow	13.9	68.33	107	–
*dfrA17/dfrAxx§*	Fast	16.4	8.5	11.39	–

*Maximum growth rate for each strain grown in the absence of T_64_.

†∆AUC calculated as AUC_T_0_ – AUC_T_64_.

§Case examples included for comparison.

In contrast, *dfrA5:dfrA7* exhibited a growth behaviour switch from a slow phenotype to a fast (Table 1). As *dfrA5:dfrA7* impacts the parental *dfrA5* phenotype, there is consistency with other alleles tested. Finally, while the *dfrA5:dfrA1* recombinant was accurate, growth assays suggested an exaggerated ∆lag_SLW_ phenotype (Table 1). This phenotype, while not expected, is still consistent with the observed growth behaviour being dependent on the *dfrA* allele carried.

### Expression analysis of *dfrA*

**Fig. 6.**
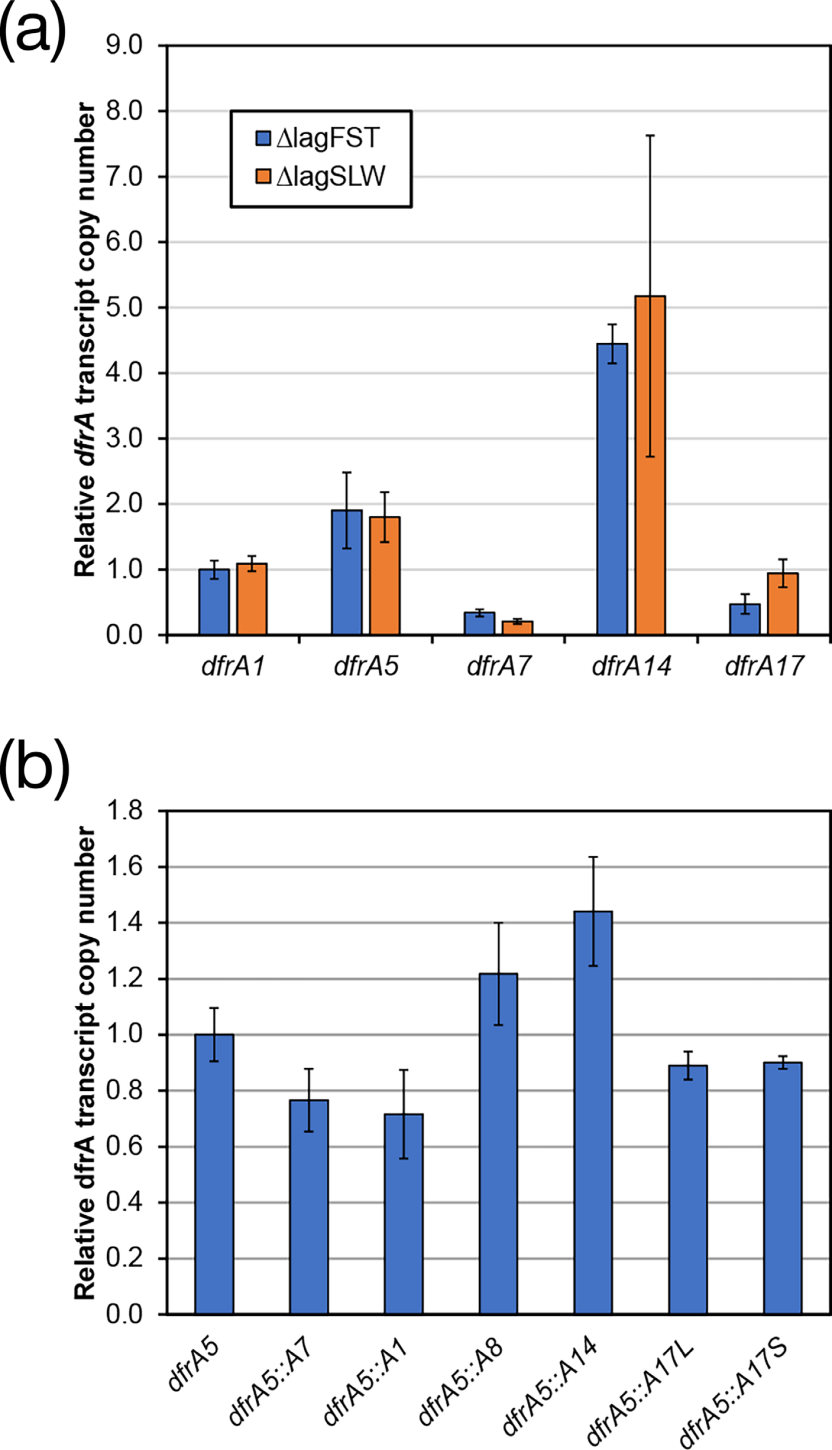
Expression analysis suggests that the growth phenotype is not dependent on changes in gene expression. The transcript copy number was determined using RT-qPCR from RNA isolated for each strain using DNA-based standard curves. (**a**) Relative transcription measured against the *dfrA1* ∆lag_FST_ allele being set at 1.0. (**b**) Relative transcription as measured against the parent *dfrA5* transcription. All data represents the average of three biological and two technical repeats of each qPCR assay.

One explanation for the difference in growth behaviour is the basal level of expression of each allele in their respective strain backgrounds. This hypothesis would also explain the phenotype of *dfrA5:dfrA1*. The transcription of isolates carrying *dfrA1*, *dfrA5*, *dfrA7*, *dfrA14* and *dfrA17* that exhibit either phenotype was quantified by calculating the transcript copy number via RT-qPCR. For *dfrA7*, specifically, the ∆lag_SLW_ strain used was the case example *dfrA7*^+^. The relative transcript copy number of each allele was compared with *dfrA1* ∆lag_FST_ (Fig. 6). This analysis identified no clear trend of ∆lag_SLW_ strains having less transcription than the ∆lag_FST_ comparator strain (Fig. 6a). This suggests that while there is a clear difference between alleles, with respect to transcription, expression cannot explain the growth behaviour identified.

In contrast to the two- to fivefold difference in gene expression seen for *dfrA5* and *dfrA14* in WT isolates, quantification of the relative transcript copy number in the engineered strains further argued against transcription being a driver of the growth behaviour. For example, comparable but slightly reduced levels of transcription were quantified for *dfrA5:dfrA7*, *dfrA5:dfrA1* and the two *dfrA5:dfrA17* recombinants. Likewise, *dfrA5:dfrA8* and *dfrA5:dfrA14* both exhibited a slightly raised level of transcription (Fig. 6b).

## Discussion

Trimethoprim resistance is predominantly underpinned by the acquisition of a single gene, *dfrA*, that exhibits a remarkable level of diversity. Using longitudinal isolates from two clinical trials has allowed us to measure the impact of long-term antibiotic use on antimicrobial resistance [9,10]. Our focus was characterizing Tri^R^ in these isolates. Our investigation has identified a slow growth behaviour that exhibits a given bias to specific *dfrA* alleles, predominantly *dfrA5*, *dfrA7* and *dfrA14*. Using a *dfrA5* ∆lag_SLW_ isolate as a parental background, we have shown that this phenotype is driven by *dfrA*.

During a structure-function analysis, Krucinska *et al.* [7] compared the activity of DfrA1 to DfrA5 and the impact of trimethoprim inhibition. This analysis defined that the K_M_ for dihydrofolate reduction to tetrahydrofolate for each enzyme was 9.7 µM compared with 24.2 µM, respectively. Furthermore, the K_i_ for trimethoprim was defined as DfrA1 : 1332 nM versus DfrA5 : 394 nM^7^. This suggests that DfrA5 is a less efficient DHFR enzyme leading to a fitness cost when exposed to trimethoprim in strains carrying this allele in comparison with others such as DfrA1. It will be of interest for future studies to generate similar kinetic data for each or all known *dfrA* alleles. For example, we can only infer that while *dfrA5* and *dfrA14* are closely related (Fig. 5), the ∆lag_SLW_ phenotype for these alleles is the result of similar properties compared with DfrA5.

The diversity amongst *dfrA* alleles identified in our strain collection reflects the level of community-based Tri^R^ and *dfrA* carriage in previous surveillance studies. Carter *et al*. [25] have characterized antibiotic resistance in 199 clinical isolates from UTI cases in 1 region of the UK [25]. The distribution of *dfrA* alleles is consistent with our data, with *dfrA1*, *dfrA5*, *dfrA12*, *dfrA14* and *dfrA17* being the predominant alleles. *dfrA5* or *dfrA14* carriage was 18.9% in Carter *et al.* [25], while we found 23%. Furthermore, Carter *et al*. [25] also identified double *dfrA* carriage: *dfrA1*/*dfrA14* and *dfrA1*/*A12* combinations. In contrast, we found *dfrA1*/*dfrA5*, *dfrA1*/*dfrA17* and *dfrA5*/*dfrA36* combinations. A common trend here is that the majority of these have *dfrA1*, which is more frequently a chromosomally encoded *dfrA* allele, while the others are on plasmids (Fig. 3).

Our screen identified that the growth behaviour was not restricted to dual carriage of *dfrA* alleles; however, all strains that did carry two copies of *dfrA* had the ∆lag_FST_ phenotype. We can attribute this to the carriage of an efficient enzyme, i.e. DfrA1, in the majority of incidences. For the *dfrA5*/*dfrA36* strain, this can further suggest that the efficacy of DfrA36 is comparable with DfrA1, or the carriage of two inefficient enzymes can act together to generate the ∆lag_FST_ phenotype. Furthermore, assessing *dfrA36* single carriers would agree with this assumption as they exhibit the ∆lag_FST_ phenotype (Table S4). Importantly, this data does suggest that dual carriage can provide strains with a fitness advantage when exposed to trimethoprim.

Whether there are any clinical implications of the observed growth phenotype will require further downstream studies targeting patient cohorts where trimethoprim use is recommended, especially as there was lower power with respect to patient numbers in ALTAR and AnTIC to correlate patient outcome and the observed phenotypes. Our definition of the observed phenotype has focussed on the lag time it takes isolates to grow when challenged with trimethoprim. *E. coli* requires a given growth rate to maintain itself in the host bladder [26]. Our data argues that the observed delay may impact the ability of *E. coli* to maintain itself in the bladder. This would potentially have implications for individuals with a *dfrA5* or *dfrA14* Tri^R^ *E. coli* infection responding to treatment. To determine whether this is true, however, requires short- and long-term monitoring of patient cohorts.

When stratifying the data with respect to the source of the isolate, it was observed that urine isolates showed a trend towards the ∆lag_SLW_ phenotype. Furthermore, for patients known to be on trimethoprim prophylaxis only, ∆lag_FST_ isolates were found in swab samples. This suggests that trimethoprim may promote the selection of ∆lag_FST_ carriage in the gut flora. These data further support the argument that prolonged antibiotic use in combination with accurate monitoring of isolated uropathogens can or should aid future clinical decisions on treatment. In conclusion, we have identified a means to differentiate between *dfrA* alleles based on their response to challenge with trimethoprim. The potential clinical impact of these findings now requires addressing.

## Supplementary material

10.1099/jmm.0.002021 Uncited Supplementary Material 1.

10.1099/jmm.0.002021 Uncited Supplementary Material 2.

## Abbreviations

DHFR: dihydrofolate reductase
MLST: multilocus sequence typing
ST: sequence type
UTI: urinary tract infection
