# Supplementary material for: Trimethoprim resistance in Escherichia coli exhibits an allele-specific growth advantage
Source: J Med Microbiol. 2025 Jun 3;74(6):002021. doi: 10.1099/jmm.0.002021 (PMC12452031; doi:10.1099/jmm.0.002021)
Supplement: Uncited Supplementary Material 1. [file jmm-74-02021-s001.pdf]

```
                  ....:...10...:...20...:...30...:...40...:...50...:...60
GAN0996_03555     M-KMIAAVGRNYEIGIANELPWRCSTDLKLFKRLTKNATVVMGRKTMESLK-RPLPERHN
GAT9054_05142     M-KMIAAVGRNYEIGIANELPWRCSTDLKLFKRLTKNATVVMGRKTMESLK-RPLPERHN
DfrA4             MIRMILAINNQCFIGKNNTLMYRLKDDMLNFKKMTQNNIVVMGRKTFESLNNRGLPNRLN
UAN8530_03343     M-KMIAAVGRNYEIGIANELPWRCSTDLKLFKRLTKNATVVMGRKTMESLK-RPLPERHN
UAN5090_04170     M-KMIAAVGRNYEIGIANELPWRCSTDLKLFKRLTKNATVVMGRKTMESLK-RPLPERHN
UAN4411_03599     M-KMIAAVGRNYEIGIANELPWRCSTDLKLFKRLTKNATVVMGRKTMESLK-RPLPERHN

                  ....:...70...:...80...:...90...:..100...:..110...:..120
GAN0996_03555     LVLTR------------------SHGFVP-------------NGFYPAGVDDVLRLPEPV
GAT9054_05142     LVLTR------------------SHGFVP-------------NGFYPAGVDDVLRLPEPV
DfrA4             VVVTSKAETFEDIQTITTHDMKRSETFTKEGHVVYITPDSFINQFLPFHRD----SEDEI
UAN8530_03343     LVLTR------------------SHGFVP-------------NGFYPAGVDDVLRLPEPV
UAN5090_04170     LVLTR------------------SHGFVP-------------NGFYPAGVDDVLRLPEPV
UAN4411_03599     LVLTR------------------SHGFVP-------------NGFYPAGVDDVLRLPEPV

                  ....:..130...:..140...:..150...:..160...:..170...:..180
GAN0996_03555     WVIGGEQIYSLFMPHVEEIWLSHIGVDVPNAD----------------------------
GAT9054_05142     WVIGGEQIYSLFMPHVEEIWLSHIGVDVPNAD----------------------------
DfrA4             WVIGGAQVYEAATPFASEIICTFVDDDEVGDVALKPKLFGGFTHLATLKSVDVDEDNDKP
UAN8530_03343     WVIGGEQIYSLFMPHVEEIWLSHIGVDVPNAD----------------------------
UAN5090_04170     WVIGGEQIYSLFMPHVEEIWLSHIGVDVPNAD----------------------------
UAN4411_03599     WVIGGEQIYSLFMPHVEEIWLSHIGVDVPNAD----------------------------

                  ....:..190...:..200...:..210...:..220...:..230...:..240
GAN0996_03555     ------------------------------------------------AFFPASMMRNLG
GAT9054_05142     ------------------------------------------------AFFPASMMRNLG
DfrA4             YEITQLVRHEDLEHKLRELQAQQHEMEKEQTQNNLSTPLENGGLRQGEAFVIAAT-TSAA
UAN8530_03343     ------------------------------------------------AFFPASMMRNLG
UAN5090_04170     ------------------------------------------------AFFPASMMRNLG
UAN4411_03599     ------------------------------------------------AFFPASMMRNLG

                  ....:..250...:..260...:...
GAN0996_03555     FVPVETVFTQRASEEEPGFSQI-VYRRS
GAT9054_05142     FVPVETVFTQRASEEEPGFSQI-VYRRS
DfrA4             LSQIDTESREDSSDSDSSSSDSSSSSSD
UAN8530_03343     FVPVETAYTQRASEEEPGFSQI-VYRRS
UAN5090_04170     FVPVETAYTQRASEEEPGFSQI-VYRRS
UAN4411_03599     FVPVETAYTQRASEEEPGFSQI-VYRRS
```

Figure S1: Alignment of DfrA4 to related alleles. Two regions of strong similarity, shown with red bars, explains the inclusion of these proteins when identifying DfrA proteins within an in-house database of all annotated proteins from the 278 AnTIC/ALTAR genomes sequenced.

```
                               <- xerC
          ....:...10....:...20....:...30....:...40....:...50....:...60
dfrA5     ACGCAGCGGTGGTAACGGCGCAGTGGCGGTTTTCATGGCTTGTTATGACTGTTTTTTTGT
dfrA7     ACGCAGCGGTGGTAACGGCGCAGTGGCGGTTTTCATGGCTTGTTATGACTGTTTTTTTGT
dfrA12    ACGCAGCGGTGGTAACGGCGCAGTGGCGGTTTTCATGGCTTGTTATGACTGTTTTTTTGT
dfrA17    ACGCAGCGGTGGTAACGGCGCAGTGGCGGTTTTCATGGCTTGTTATGACTGTTTTTTTGT
dfrA14    ACGCAGCGGTGGTAACGGCGCAGTGGCGGTTTTCATGGCTTGTTATGACTGTTTTTTTGT

          ....:...70....:...80....:...90....:..100....:..110....:..120
dfrA5     ACAGTCTATGCCTCGGGCATCCAAGCAGCAAGCGCGTTACGCCGTGGGTCGATGTTTGAT
dfrA7     ACAGTCTATGCCTCGGGCATCCAAGCAGCAAGCGCGTTACGCCGTGGGTCGATGTTTGAT
dfrA12    ACAGTCTATGCCTCGGGCATCCAAGCAGCAAGCGCGTTACGCCGTGGGTCGATGTTTGAT
dfrA17    ACAGTCTATGCCTCGGGCATCCAAGCAGCAAGCGCGTTACGCCGTGGGTCGATGTTTGAT
dfrA14    ACAGTCTATGCCTCGGGCATCCAAGCAGCAAGCGCGTTACGCCGTGGGTCGATGTTTGAT

          ....:..130....:..140....:..150....:..160....:..170....:..180
dfrA5     GTTATGGAGCAGCAACGATGTTACGCAGCAGGGCAGTCGCCCTAAAACAAAGTTAACCCG
dfrA7     GTTATGGAGCAGCAACGATGTTACGCAGCAGGGCAGTCGCCCTAAAACAAAGTTAGCCAT
dfrA12    GTTATGGAGCAGCAACGATGTTACGCAGCAGGGCAGTCGCCCTAAAACAAAGTTAGCCAT
dfrA17    GTTATGGAGCAGCAACGATGTTACGCAGCAGGGCAGTCGCCCTAAAACAAAGTTAGCCAT
dfrA14    GTTATGGAGCAGCAACGATGTTACGCAGCAGGGCAGTCGCCCTAAAACAAAGTTAACCCA

          ....:..190....:..200....:..210....:..220....:..230....:..240
dfrA5     G--AACCAA--AATTGTGAAAGTATCATTAATGGCTGCAAAAGCGAAAAACGGAGTGATT
dfrA7     T--ACGGGGGTTGAATTGAAAATTTCATTGATTTCTGCAACGTCAGAAAATGGCGTAATC
dfrA12    ATGAACTCGGAATCAGTACGCATTTATCTCGTTGCTGCGATGGAGCCAATCGGGTTATT
dfrA17    T--AAGGGAGTTAAATTGAAAATATCATTGATTTCTGCAGTGTCAGAAAATGGCGTAATC
dfrA14    G--GATGAG--AACCTTGAAAGTATCATTGATGGCTGCGAAAGCGAAAAACGGCGTGATT

          ....:..250....:..260....:..270....:..280....:..290....:..300
dfrA5     GGTTGCGGTCCACACATACCCTGGTCCGCGAAAGGAGAGCAGCTACTCTTTAAAGCCTTG
dfrA7     GGTAATGGCCCTGATATCCCATGGTCAGCAAAAGGTGAGCAGTTACTCTTTAAAGCGCTC
dfrA12    GGCAATGGTCCTAATATCCCCTGGAAAATTCCGGGTGAGCAGAAGATTTTTCGCAGACTC
dfrA17    GGTAGTGGTCCTGATATCCCGTGGTCAGTAAAAGGTGAGCAACTACTCTTTAAAGCGCTC
dfrA14    GGTTGCGGTCCAGACATACCCTGGTCCGCGAAAGGGGAGCAGCTACTTTTTAAAGCATTG

          ....:..310....:..320....:..330....:..340....:..350....:..360
dfrA5     ACGTACAACCAGTGGCTTTTGGTGGCCGCAAGACGTTCGAATCTATGGGAGC---ACTC
dfrA7     ACATATAATCAGTGGCTCCTTGTTGGAAGGAAAACATTTGACTCTATGGGTGT---TCTT
dfrA12    ACTGAGGGAAAAGTCGTTGTCATGGGCGAAAGACCTTTGAGTCTATCGGCAAGCCTCTA
dfrA17    ACATATAATCAATGGCTCCTTGTCGGAAGAAAAACATTTGACTCTATGGGTGT---TCTT
dfrA14    ACCTACAATCAGTGGCTTCTGGTGGGTCGCAAGACGTTTGAATCTATGGGCGC---ACTC
```

Figure S2: DNA sequence similarity identified upstream of the annotated start codons for *dfrA5*, *dfrA7*, *dfrA12*, *dfrA17* and *dfrA14* in AnTIC /ALTAR isolates. The start codon for *xerC* is indicated in red. The alternative start codon often used during PROKKA annotation of genomes with *dfrA7* and *dfrA17* is shown in yellow. The defined start codon of reference genes for each allele are shown in green.

A.

```
                 ....:...10....:...20....:...30....:...40....:...50....:...60
DfrA7_04879      MRSRNWSRTLTERSGGNGAVAVFMACYDCFFVQSMPRASKQQARYAVGRCLMLWSSNDVT
DfrA17           ------------------------------------------------------------
DfrA17_04990     ------------------------------------------------------------
DfrA17_04834     ---------------------------------------------------MLWSSNDVT
DfrA7            ------------------------------------------------------------
DfrA17_04972     ----------------------------------MPRASKQQARYAVGRCLMLWSSNDVT
DfrA17_04802     MRSRNWSRTLTERSGGNGAVAVFMACYDCFFVQSMPRASKQQARYAVGRCLMLWSSNDVT
DfrA7_04461      ---------------------------------------------------MLWSSNDVT
DfrA17_04946     ---------------------------------------------------MLWSSNDVT
DfrA17_04420     ----------------------------------MPRASKQQARYAVGRCLMLWSSNDVT

                 ....:...70....:...80....:...90....:..100....:..110....:..120
DfrA7_04879      QQGSRPKTKLAITGVELKISLISATSENGVIGNGPDIPWSAKGEQLLFKALTYNQWLLVG
DfrA17           ----------------MKISLISAVSENGVIGSGPDIPWSVKGEQLLFKALTYNQWLLVG
DfrA17_04990     ----------------MKISLISAVSENGVIGSGPDIPWSVKGEQLLFKALTYNQWLLVG
DfrA17_04834     LQGSRPKTKLAIKGVKLKISLISAVSENGVIGSGPDIPWSVKGEQLLFKALTYNQWLLVG
DfrA7            ----------------MKISLISATSENGVIGNGPDIPWSAKGEQLLFKALTYNQWLLVG
DfrA17_04972     QQGSRPKTKLAIKGVKLKISLISAVSENGVIGSGPDIPWSVKGEQLLFKALTYNQWLLVG
DfrA17_04802     QQGSRPKTKLAIKGVKLKISLISAVSENGVIGSGPDIPWSVKGEQLLFKALTYNQWLLVG
DfrA7_04461.     QQGSRPKTKLAITGVELKISLISATSENGVIGNGPDIPWSAKGEQLLFKALTYNQWLLVG
DfrA17_04946     QQGSRPKTKLAIKGVKLKISLISAVSENGVIGSGPDIPWSVKGEQLLFKALTYNQWLLVG
DfrA17_04420     QQGSRPKTKLAIKGVKLKISLISAVSENGVIGSGPDIPWSVKGEQLLFKALTYNQWLLVG
```

B.

```
                  ....:...10....:...20....:...30....:...40....:...50....:...60
DfrA5:A7_out      MPRAS---KQQARYAVGRCLMLWSSNDVTQQGSRPKTKLLMLWSSNDVTQQGSRPKTKLA
DfrA5:A17_S       ------------------------------------------------------------
DfrA5:A7_dsgn     ----------------------------------------MLWSSNDVTQQGSRPKTKLA
DfrA5:A17_L       MPRAS---KQQARYAVGRCL--------------------MLWSSNDVTQQGSRPKTKLA
DfrA5:A17_dsgn_L  MPRAS---KQQARYAVGRCL--------------------MLWSSNDVTQQGSRPKTKLA

                  ....:...70....:...80....:...90....:..100....:..110....:..120
DfrA5:A7_out      ITGVELKISLISATSENGVIGNGPDIPWSAKGEQLLFKALTYNQWLLVGRKTFDSMGVLP
DfrA5:A17_S       ----MKKISLISAVSENGVIGSGPDIPWSVKGEQLLFKALTYNQWLLVGRKTFDSMGVLP
DfrA5:A7_dsgn     ITGVELKISLISATSENGVIGNGPDIPWSAKGEQLLFKALTYNQWLLVGRKTFDSMGVLP
DfrA5:A17_L       IKGVKLKISLISAVSENGVIGSGPDIPWSVKGEQLLFKALTYNQWLLVGRKTFDSMGVLP
DfrA5:A17_dsgn_L  IKGVKLKISLISAVSENGVIGSGPDIPWSVKGEQLLFKALTYNQWLLVGRKTFDSMGVLP
```

Figure S3: Predicted peptide sequences for the N-terminus of DfrA7 and DfrA17. **A.** The summary of alternative annotations observed when running PROKKA annotation on *dfrA7*$^{+}$ and *dfrA17*$^{+}$ genomes compared to the defined reference sequence of DfrA17 and DfrA17. **B.** Predicated N-terminus regions of DfrA5:A(x) recombinants. DSGN: the expected sequence; -out: the predicted peptide sequence of the DfrA5:A7 recombinant.

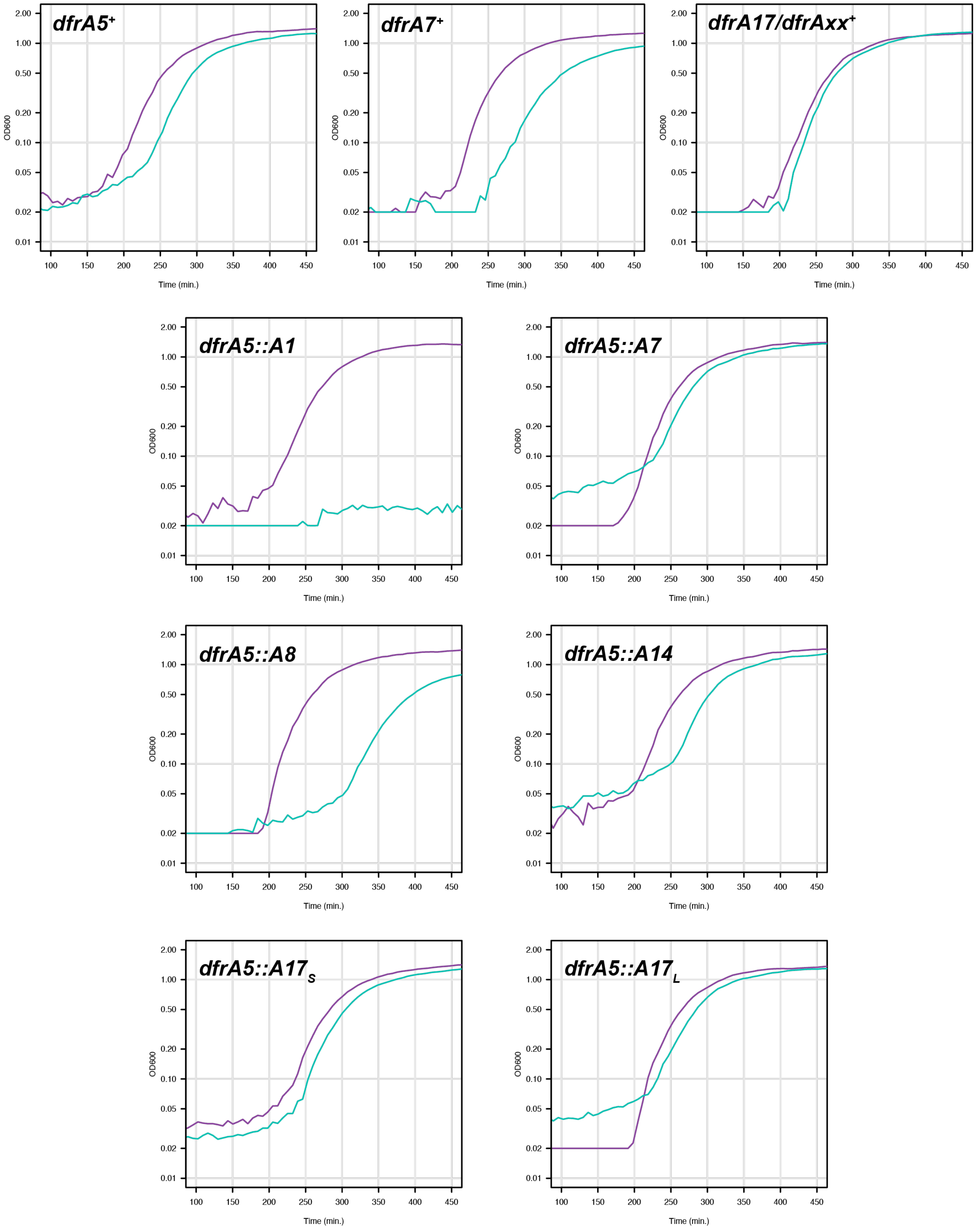

Figure S4: Growth phenotypes of the recombinant dfrA5::(x) alleles compared to the parent *dfrA5*$^{+}$ and the controls *dfrA7* and *dfrA17/dfrAxx*$^{+}$. All data represents the average of a minimum of 3 independent biological repeats, with error bars omitted for clarity. Growth without trimethoprim is shown in purple and with 64 mg/L trimethoprim in teal. Growth analysis is shown in Table 1.
